# Effects of M-DEPTH model of depression care on maternal depression, functioning, and HIV care adherence, and infant developmental over eighteen months post-partum: results from a cluster randomized controlled trial

**DOI:** 10.1186/s12884-025-07443-0

**Published:** 2025-04-05

**Authors:** Glenn J. Wagner, Bonnie Ghosh-Dastidar, Violet Gwokyalya, Laura J. Faherty, Jolly Beyeza-Kashesya, Juliet Nakku, Linda Kisaakye Nabitaka, Dickens Akena, Janet Nakigudde, Victoria Ngo, Ryan McBain, Hafsa Lukwata, Leticia Kyohangirwe, Barbara Mukasa, Rhoda K. Wanyenze

**Affiliations:** 1 RAND Corporation, 1776 Main Street, Santa Monica, CA 90407 USA; 2 School of Public Health, Makerere University, Kampala, Uganda; 3 Boston University School of Medicine, Boston, MA USA; 4 Mulago Specialized Women and Neonatal Hospital, Kampala, Uganda; 5 School of Medicine, Makerere University, Kampala, Uganda; 6 Butabika National Referral Mental Hospital, Kampala, Uganda; 7 Ministry of Health, Kampala, Uganda; 8 College of Health Sciences, Makerere University, Kampala, Uganda; 9 Graduate School of Public Health and Health Policy, City University of New York, New York, USA; 10 Mildmay Uganda, Kampala, Uganda

**Keywords:** Depression, HIV, Problem solving therapy, Antidepressant therapy, Maternal functioning, Child development

## Abstract

**Background:**

Perinatal depression is associated with poor outcomes related to HIV care adherence, maternal functioning, and early child development. We examined whether the M-DEPTH (Maternal Depression Treatment in HIV) depression care model—including antidepressant therapy and individual problem-solving therapy—and depression alleviation would affect improvement in each of these outcome domains.

**Methods:**

A sample of 354 pregnant women living with HIV (WLH) with at least mild depressive symptoms (177 in each of intervention and usual care control arms) enrolled in a cluster randomized controlled trial across eight antenatal care clinics in Uganda and had a live birth delivery. Longitudinal mixed effects models were used to examine survey data and chart-abstracted HIV viral load and antiretroviral pharmacy refill data collected at baseline and months 2, 6, 12 and 18 post-partum.

**Results:**

69% had clinical depression at enrollment; 70% of women in the intervention group (including 96% of those with clinical depression) received depression treatment. Mixed-effects longitudinal regression analysis showed (1) strong effects of the intervention on maternal depression at each post-partum follow-up assessment; and (2) moderate effects of the intervention and reduced depression on maternal functioning (self-care and infant care, in particular). However, there was little evidence of effects of the intervention and depression reduction on early child development, maternal viral suppression, or ART adherence.

**Conclusion:**

These findings suggest that depression care for pregnant WLH is important for maternal mental health, but it also helps women to better manage parenting and care for their infant. Supplementary interventions may be needed to impact early child development.

**Trial registration:**

The trial was registered with the NIH Clinical Trial Registry (clinicaltrials.gov: NCT03892915) on 27/03/2019.

**Supplementary Information:**

The online version contains supplementary material available at 10.1186/s12884-025-07443-0.

## Background

Depression is common during pregnancy or post-partum among women world-wide [1, 2], including women in sub-Saharan Africa [3]. Similar or slightly higher rates have been documented among pregnant women living with HIV (WLH) in sub-Saharan Africa, where 30–50% of such women suffer elevated depressive symptoms and about one-third have a depressive disorder [4–6]. Task-shifted, collaborative care models, in which specialists train and supervise lay and medical staff to carry out processes of depression care, demonstrate cost-effectiveness in delivering evidence-based depression care in low-resource settings [7–9], including Problem Solving Therapy (PST) [10] and antidepressant therapy (ADT) [11, 12] in sub-Saharan Africa. Similar depression care models have been effective for perinatal depression in the United States [13, 14], but have not been studied among pregnant or post-partum women in sub-Saharan Africa or WLH.

**Fig. 1:**
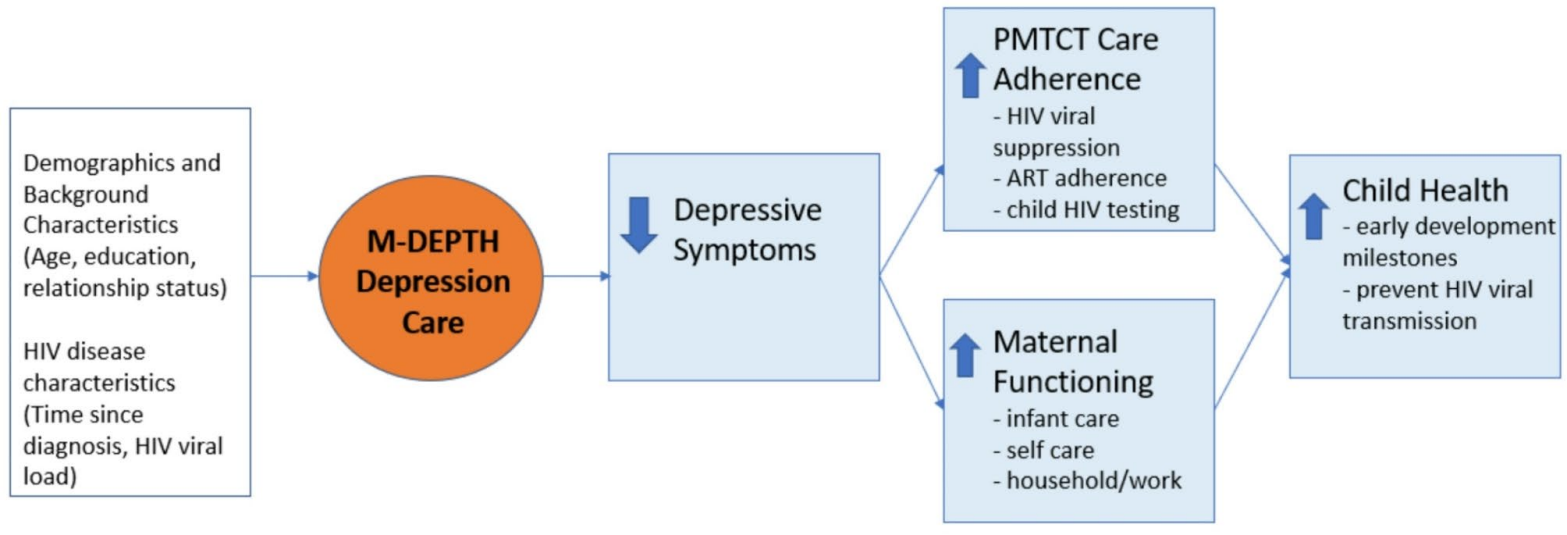
Conceptual framework for the effects of M-DEPTH and reduced depression on maternal functioning, PMTCT care adherence, and child health outcomes

Perinatal depression can impair the mother’s ability to properly care for herself and her child, resulting in adverse effects on maternal functioning and parenting, as well as early child health and development [15–17]. For WLH, perinatal depression has the added consequences of reduced viral suppression [18] and poor use of and adherence to HIV antiretroviral therapy [19], which increases the risk of vertical transmission of HIV to the newborn [20]. Effective treatment for depression may be expected to ameliorate these adverse effects and improve maternal and child health outcomes (see Fig. 1). Depression alleviation may improve a mother’s ability and motivation to function and care for herself (e.g., use proper nutrition, get proper sleep), manage her HIV disease and pregnancy [e.g., attend antenatal care visits, adhere to HIV antiretroviral therapy (ART) and other aspects of the prevention of mother-to-child transmission (PMTCT) care continuum], and engage in effective parenting interactions post-partum.

Controlled trials of treatment for perinatal depression have shown benefits for maternal functioning and parenting [21–23], but findings have been mixed for effects on child outcomes [24, 25]. Research on these maternal and child effects of maternal depression treatment in the context of sub-Saharan Africa is scarce. In the context of HIV, studies of depression treatment have largely been conducted in the context of general HIV care, not antenatal care or PMTCT; some results suggest that depression treatment improves ART use and adherence [26, 27], but others have shown only effects on depression [28]. 

To begin to fill these gaps in the literature, Maternal Depression Treatment in HIV (M-DEPTH) -- a cluster randomized controlled trial of a collaborative care model of evidence-based treatment for perinatal depression among women living with HIV—was recently conducted across eight public health antenatal care (ANC) facilities in Uganda [29]. Participants with clinical depression were treated with PST or ADT, and participants were followed from pregnancy through 18 months post-partum. Prior publications have documented intervention effects through six months post-partum. At this early stage of follow-up, the depression care model was highly efficacious for depression alleviation, with those in the intervention arm 80% less likely to be depressed compared to those in the control group, and high response rates to both PST (83%) and ADT (67%) [30, 31]. There were also benefits for maternal functioning (parenting and self-care, specifically) [32], but no effects were observed for maternal HIV viral suppression, ART adherence, or early child development outcomes [30].

With the study now complete, this paper reports findings from data that spanned the full 18-month post-partum follow-up. The primary objectives of the analysis were to examine (1) the effects of the intervention on depression, HIV viral suppression and ART adherence, maternal functioning, and child development; and (2) the effects of depression alleviation (i.e., reduced depressive symptoms, and depression remission) on these same outcomes.

## Methods

### Study design

This study is a cluster randomized controlled trial of an evidence-based, collaborative care model of depression care for pregnant WLH in Uganda. The study was performed at eight ANC clinics within public health facilities operated by the Ministry of Health, with technical assistance from Mildmay Uganda for HIV reproductive health; four were randomly assigned to implement the intervention in addition to usual care, and four to implement usual care alone. Providers, participants, and data collectors were unblinded to the assignment of the treatment condition; only the data analyst was blinded. Assessments were conducted at baseline and months 2, 6, 12 and 18 post-partum (except for women who did not have a successful delivery; their last assessment was month 6 post-partum). Participants received anywhere from 20,000 Ush (~$6 USD) to 50,000 Ush (~$15 USD) to cover transportation costs for attending study assessment visits, depending on the distance they needed to travel; 20,000 Ush was provided for attending each treatment visit. The study protocol was reviewed and approved by institutional review boards at the RAND Corporation and Makerere University School of Public Health, and the Uganda National Council for Science and Technology. CONSORT guidelines were followed in reporting the study findings and a CONSORT checklist is available as a supplementary file.

### Participants

The target sample size was 50 participants per site, based on a sample size calculation that set power at 80% (alpha = 0.05; two-tailed test) to detect a 7% difference between the two study arms (small effect size; Cohen’s d = 0.16) with regards to achievement of undetectable HIV viral load (the primary outcome of the study) at month 2 post-partum (assuming an intra-cluster correlation coefficient of 0.01, 10% attrition at month 2 post-partum, and a 65% undetectable viral load rate in the control group) [30]. 

Recruitment took place between July 2019 and January 2021. Eligibility criteria for women attending the ANC clinic included: (1) gestation period of no more than 32 weeks, (2) HIV-positive (confirmed by medical provider), (3) age 18 years or older, and (4) scored > 4 on the 9-item Patient Health Questionnaire (PHQ-9) [33], which indicates at least mild depressive symptoms [30, 32]. At each site, all adult clients living with HIV who were early enough in their pregnancy to be eligible were screened for potential depression by trained peer mothers using the two-item PHQ-2 at routine clinic visits [30, 32]. Those who screened positive (i.e., a score greater than zero) received depression psychoeducation and were informed of the study; those who expressed interest in participating were further evaluated by a midwife nurse who administered the PHQ-9. Women who scored > 4 on the PHQ-9, were medically stable (i.e., no acute opportunistic infections and were on ART for at least 4 weeks), not considered to be at high risk of suicide, and not currently receiving mental health treatment, were referred to the study coordinator to confirm eligibility and consent procedures. These screening and evaluation processes of determining eligibility were also the initial depression screening and treatment eligibility evaluations of the depression care model, as described below. All women who enrolled provided written informed consent [30, 32]. 

### Treatment conditions

#### Usual care

Usual care procedures for addressing depression in the participating ANC clinics were limited to referral of patients exhibiting severe depressive symptoms to a mental health specialist (typically a psychiatric nurse), either at the facility or the nearest District or Regional Referral Hospital. In addition, each study site offered Family Support Groups (FSG) to clients living with HIV to provide education and psychosocial support for pregnancy and PMTCT management. The FSG program consisted of monthly group sessions from the antenatal phase through 18 months post-partum.

#### M-DEPTH depression care

Drawing from evidence-based collaborative care models for depression [7, 34], the primary components of the depression care model consisted of (1) *depression screening* implemented by peer mothers using the PHQ-2; (2) *evaluation of treatment eligibility*, including positive PHQ-9 screens by the midwife nurse; (3) *depression psychoeducation* and recommendation of treatment modality by the midwife nurse [PST for moderate depression (PHQ-9 = 10–19) and ADT for severe depression (PHQ-9 > 19)]; and (4) *provision of treatment* [individual PST (up to seven bi-weekly sessions) [35] or ADT (first-line medication was fluoxetine, and treatment continued until 6 months in remission)]. Participants with minor depression (PHQ-9 = 5–9) received psychoeducation and continued depression monitoring at monthly usual care visits. Peer mothers and midwife nurses on staff at the clinics were trained during a 3-day workshop and received ongoing supervision from mental health specialists hired by the study. A more detailed description of the model is available in a prior publication [29]. 

### Measures

Assessments included a computer-assisted interviewer-administered survey, laboratory assays, pharmacy data, and data abstracted from medical charts and the Depression Care Registry. Survey measures that had not been translated into Luganda during our prior research were translated using standard translation, back-translation methods. All measures were collected at each assessment, except for maternal functioning and child development measures, which were only assessed at the post-partum follow-up assessments (not at baseline).

#### Adherence to PMTCT care continuum

These measures included the following: ART use and adherence [percent of prescribed pills taken: (pills dispensed/pills prescribed per day)/days between refills, multiplied by 100), based on pharmacy refill data]. Analysis used a binary variable representing good ART adherence, defined as refill data indicating no gaps in medication access (i.e., 100% adherence). Retention in ANC care was assessed through abstracted clinic attendance data. Infant HIV testing (at month 18) and serostatus was ascertained via chart abstraction. Maternal virologic suppression was assessed with HIV viral load tests performed by the study at enrollment and the month 2 post-partum assessment; viral load results from tests conducted as part of usual care through month 18 were abstracted from clinical records. Analysis used a binary variable representing undetectable (< 20 copies/mm^3^) viral load, and log_10_ viral load change from baseline.

#### Maternal functioning

We used four subscales of the Inventory of Functional Status After Childbirth to measure these aspects of post-partum maternal functioning: infant care responsibilities (6 items), self-care activities (8 items), household activities (12 items), and work-related activities (4 items) [36]. Each item has a response range of 1 to 4, excluding a ‘not applicable’ response. The mean item score for each subscale was calculated, as well as the mean of all items to measure overall functioning; higher scores indicate better functioning. The 9-item subscale of the Postpartum Adjustment Questionnaire [37] was used to assess adjustment to the motherhood role by asking the participant’s perception of her adequacy in caring for her baby’s needs (e.g., feeding), physical contact with baby (e.g., rocking, kissing), and play activity with the baby. Each item was scored between 1 ‘better than average or optimal level of functioning’ to 5 ‘very poor functioning’; mean item was calculated, and lower scores represent better perceived adjustment to motherhood.

#### Early child development

The 30-item Ages and Stages Questionnaire [38] was used to measure five developmental areas: communication, gross motor, fine motor, problem solving, and personal-social [32]. Mothers were asked to report observed specific behaviors in their infant that reflected age-appropriate development. Each subscale is comprised of six items, with each item having three response options: 10 ‘yes’, 5 ‘sometimes’ and 0 ‘not yet’; scores are summed (range = 0–60 for each subscale), and higher scores reflect more advanced development [32]. 

#### Depression

The 9-item Patient Health Questionnaire (PHQ-9) [33] was used to assess depression. Each item corresponds to the nine symptoms assessed in the depression module of the Diagnostic Statistical Manual of Mental Disorders [39]. Each item is scored from 0 to 3 to represent frequency of symptom presence in the past two weeks, and scores > 9 (possible range: 0 to 27) represent clinical depression and have been shown to correspond highly with diagnosis of major depressive disorder [33]. The PHQ-9 has been used successfully to assess depression in sub-Saharan Africa [40]. 

#### Background characteristics

Socio-demographics that were measured included age, education level (binary indicator of any formal secondary education was used in analysis), and relationship status (binary indicator of being in a committed relationship was used in analysis). Measured HIV disease characteristics included time since HIV diagnosis and time on ART [32]. 

### Data analysis

We conducted *t*-tests and chi-squared tests to evaluate differences in distributions of participant characteristics (i.e., socio-demographics, HIV disease, depression) at baseline for the two study arms. We repeated this analysis to compare those who completed the month 18 post-partum assessment with those who did not, to assess effect of nonresponse.

The primary analysis examined the effect of the M-DEPTH intervention on depression status, viral load, good ART adherence, maternal functioning and child development outcomes across 18-month post-partum follow-up using linear mixed-effects regression for all continuous outcomes. For the two binary outcomes (undetectable viral load, good ART adherence), we used logistic mixed-effects regression. Each of the above models adjusted for the following baseline covariates: age, any secondary education, relationship status, newly diagnosed with HIV (past 3 months), undetectable HIV viral load, and depression level (PHQ-9 at screening); for the ART adherence model, we also adjusted for adherence at baseline. Time was entered as categorical to allow for non-linear effects; interactions between each time point and intervention indicator were included to assess intervention effects. We reported a joint test of the coefficients of time and a second joint test of the four interaction terms between time and intervention, conducted using a Type-3 test of fixed effects to detect a significant overall time trend and intervention effects. We also report separate coefficients and statistical significance for each interaction term (or, follow-up assessment) as the intervention effect is modeled as non-linear across time (to examine how effects change across the study time points).

The secondary analysis examined the effect of change in depression on outcomes, in addition to overall time trends in the outcome. PHQ-9 data from the current and baseline assessments were used to calculate two measures of change at each follow-up assessment. For the continuous variable (change in PHQ-9 score), the PHQ-9 score for the current assessment minus the baseline PHQ-9 score was calculated. For the categorical variable (change in depression status, with PHQ-9 > 9 defining the presence of depression), a four-level categorical variable was calculated using current and baseline status: “repeatedly depressed” (i.e., depressed at baseline and at current assessments); “repeatedly not depressed” (i.e., not depressed at both assessments); “depressed to not depressed” (i.e., depressed at baseline, not depressed at current assessment) and “not depressed to depressed” (i.e., not depressed at baseline, depressed at current assessment).

To assess effects of change (improvement) in depression and the outcomes, we fit longitudinal models regressed on either change in PHQ-9 sore or change in depression status from the baseline to the current post-partum follow-up assessment, with *repeatedly depressed* serving as the reference category. Changes in depression status were only observable at the follow-up assessments; therefore, this analysis modeled outcome information from the corresponding assessments. Specifically, we fit linear mixed effects models for the continuous outcomes and logistic mixed effects models for the binary outcomes including baseline value (PHQ-9 score or depression status) and change from baseline, controlling for covariates. In this analysis, we combined the study arms because we observed change in depression across women in both study arms. Due to this approach, the study design is observational rather than a randomized trial. To help with assessment of practical significance, we have included effect sizes for change in depression using appropriate formulae depending on whether the model used the continuous or binary depression change measure [41, 42]. 

The mixed effects models included both fixed effects and random effects to account for clustering. Additionally, this type of modeling allows inclusion of all available observations even when individuals are missing one or more assessments. A maximum likelihood approach is taken to handle missing data. Given the very low rates of item nonresponse at each of the follow-up assessments (i.e., no more than 5%), we chose not to use weights or imputation to account for missing data. To account for multiplicity, we adjusted the significance level by number of outcomes within each domain (e.g., 6 outcomes for maternal functioning; 0.05/6 = 0.008 *p* value needed for significance). Analyses were conducted using SAS version 9.4 (SAS Institute Inc).

## Results

### Sample characteristics

**Fig. 2:**
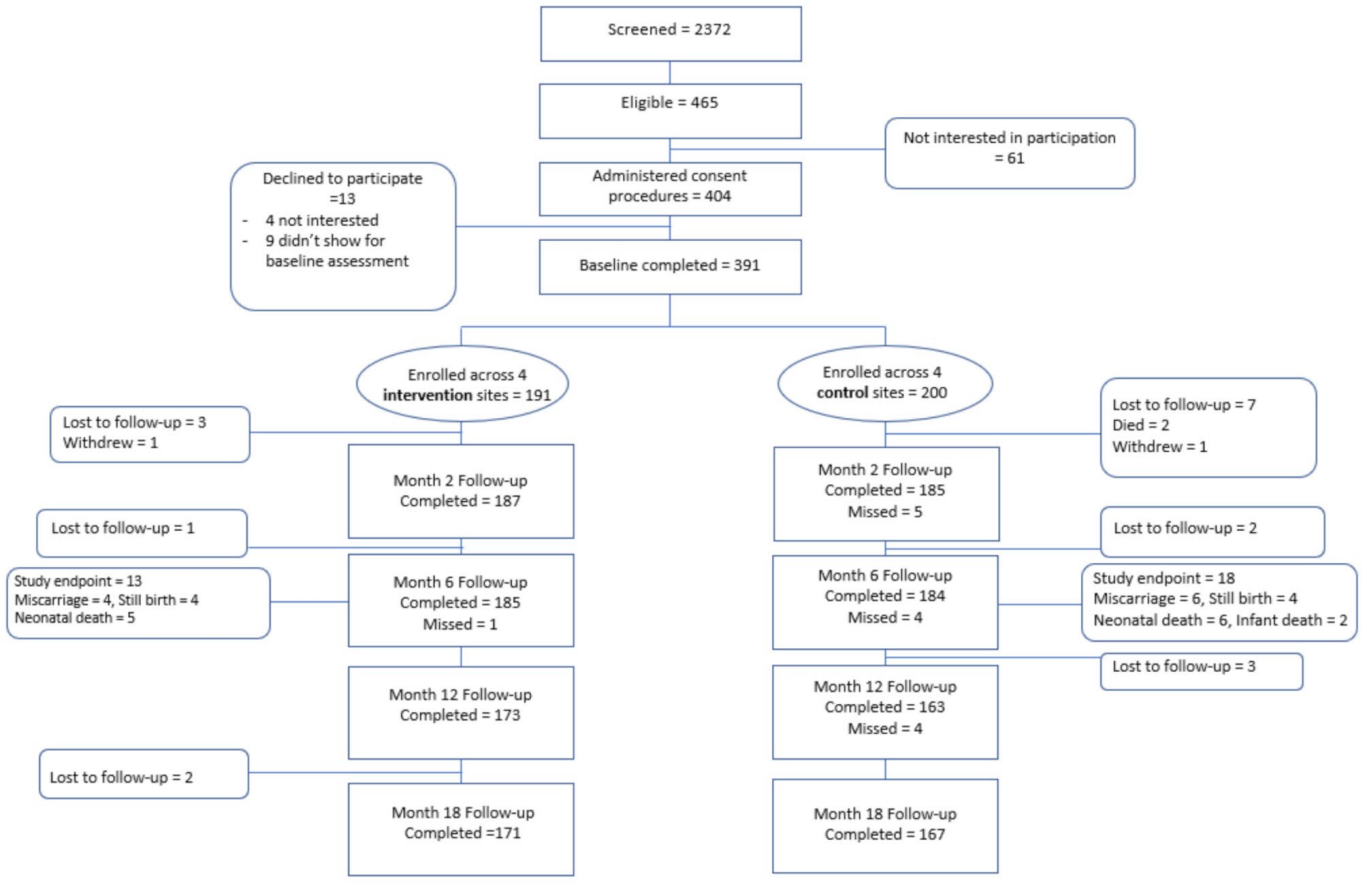
Flow of participants from screening and enrollment through 18 months post-partum follow-up

The consort diagram for flow of participants through the study protocol is depicted in Fig. 2. A total of 2,372 adult HIV-infected pregnant women were screened for study eligibility across the eight study sites; 465 were eligible, of whom 391 (84.1%) enrolled in the study (200 at control sites; 191 at intervention sites). 354 women (177 in each study arm) had live births and were followed through 18 months post-partum. Thus, the analytic sample for this analysis is 354. Table 1 lists baseline characteristics of the analytic sample. While women in the two study arms did not differ on socio-demographic characteristics, stages of HIV disease or gestation, women in the control group had higher levels of depression at baseline compared with those in the intervention group (see Table 1). Study retention was high with 338 (95.5%) women (171 intervention, 167 control) completing the month 18 assessment. Those completing the month 18 assessment were older and more likely to be in a relationship but did not differ on other baseline characteristics compared to those not completing the assessment (see Table 1).

**Table 1:** Baseline characteristics by study arm and completion of the 18-month post-partum assessment (M18), among women with a live birth (*n* = 354)

Characteristic	Total sample (*n* = 354) Mean (SD)/%	Control (*n* = 177) Mean (SD)/%	Intervention (*n* = 177) Mean (SD)/%	*p*-value	Did not complete M18 (*n* = 16) Mean (SD)/%	M18 Completers (*n* = 338) Mean (SD)/%	*p*-value
Age	27.8 (5.9)	27.9 (6.0)	27.6 (5.7)	0.72	24.8 (5.8)	27.9 (5.9)	0.04
Any secondary education	35.0%	30.5%	39.5%	0.08	31.3%	35.2%	0.75
In a committed relationship	82.5%	83.6%	81.4%	0.58	62.5%	83.4%	0.03
Gestation week	21.5 (5.9)	21.7 (5.4)	21.3 (6.4)	0.50	22.8 (5.5)	21.5 (5.9)	0.39
Months since HIV diagnosis	45.2 (43.7)	46.4 (42.6)	44.0 (44.9)	0.61	24.4 (28.6)	46.1 (44.0)	0.07
Diagnosed with HIV within past 3 mos.	21.2%	20.3%	22.0%	0.70	37.5%	20.4%	0.10
Weeks on ART at baseline	173 (177)	178 (172)	168 (184)	0.29	103 (123)	177 (179)	0.11
Undetectable HIV viral load	59.0%	59.9%	58.2%	0.75	37.5%	60.1%	0.07
PHQ-9 score	12.8 (5.2)	13.7 (5.6)	11.9 (4.5)	< 0.001	10.6 (5.2)	12.9 (5.1)	0.08
Depressed (PHQ-9 > 9)	68.9%	74.0%	63.8%	0.04	50.0%	69.8%	0.09

PHQ-9 = Patient Health Questionnaire (9-item version); ART = antiretroviral therapy; SD = standard deviation

Of the 177 women in the intervention group, 124 (70.1%) received depression treatment (82 PST, 42 ADT) -- including 96% (120/125) of those who were clinically depressed at any point during the study, and thus in need of treatment. Among all participants, we examined change in depression status (based on PHQ-9 > 9 representing depression) from baseline at each of the four post-partum follow-up assessments, for each participant. Of the 1416 potential comparisons, data were available for 1352 (95.5%); across all comparisons, 51.3% (57.7% intervention, 44.6% control) were characterized as changing from depressed to not depressed, 25.6% remained not depressed (32.3% intervention, 18.7% control), 18.3% remained depressed (7.5% intervention, 29.5% control), and 4.9% (7.3% intervention, 2.6% control) changed from not depressed to a state of depression.

### Effects on depression

The depression care intervention had a significant overall beneficial effect on both depressive symptoms [F(df) = 20.4 (4,998), *p* <.0001) and depression status [F(df) = 15.7 (4,998), *p* <.0001], across the 18-month post-partum follow-up. Furthermore, the intervention effect was statistically significant at each of the follow-up assessments for both depression measures (see Table 2).

**Table 2:** Longitudinal mixed effects models evaluating the effects of the M-DEPTH intervention on depressive symptoms (PHQ-9 score) and depression status (PHQ-9 > 9) over 18 months of post-partum follow-up

	PHQ-9 score	Depression status
Joint significance tests	F (df); *p*	F (df); *p*
Time	37.0 (3, 998); < 0.0001	7.6 (3, 998); < 0.0001
Time X intervention	20.4 (4, 998); <0.0001	15.7 (4, 998); < 0.0001
**Interaction effects**	**Beta (SE); p**	**OR (95% CI)**
Intervention X Month 2	-4.03 (0.49); <0.0001	0.21 (0.13, 0.36)
Intervention X Month 6	-3.28 (0.50); <0.0001	0.13 (0.07, 0.25)
Intervention X Month 12	-2.89 (0.50); <0.0001	0.23 (0.12, 0.43)
Intervention X Month 18	-3.40 (0.50); <0.0001	0.16 (0.08, 0.33)

SE = standard error; OR = odds ratio; CI = confidence interval; df = degrees of freedom; PHQ-9 = Patient Health Questionnaire (9-item version)

All models included these baseline covariates: age, relationship status (in a committed relationship; yes/no), any secondary schooling (yes/no), newly diagnosed with HIV (yes/no), PHQ-9 score (or depression status (yes/no) for that model) at eligibility screening, undetectable HIV viral load (yes/no). The model specification included time as a categorical variable with indicators for each follow-up assessment (month 2, month 6, month 12, month 18). The joint tests of overall effect of time and intervention are Type 3 tests of fixed effects. The coefficients of the main effects of time are not shown here. The key coefficients of interest are the interaction terms between Intervention and time, which are the (covariate-adjusted) difference between the Intervention (intervention = 1) group and the control group (Intervention = 0); a *p*-value < 0.05 indicates a statistically significant difference between the means of the two study arms. Further, note that to estimate the mean in the intervention group, one must add the main effect of time to the interaction between intervention and time

### Effects on HIV care adherence

Of the 338 women in the sample who completed month 18, 337 (99.7%) were on ART and all 338 remained in care; 306 (90.5%) women had their newborn infants tested for HIV at 18 months post-partum and none tested HIV-positive. Given the low variance in these HIV care variables, they were not examined for effects of the intervention and depression alleviation. The intervention did not have an overall effect on the two HIV viral load measures (change in log_10_ HIV RNA, undetectable HIV viral load) across the 18-month post-partum follow-up (see Table 3). Further, change in depression status and change in depressive symptoms did not have a significant effect on either outcome. However, there was a significant beneficial effect of the intervention on good ART adherence [F(df) = 4.4 (4,946); *p* =.002], although the effect was small and isolated to the 2-month post-partum assessment (see Table 3).

**Table 3:** Longitudinal mixed effects models evaluating the effects of the M-DEPTH intervention, and change in depression, on measures of HIV viral suppression and ART adherence over 18 months of post-partum follow-up

	HIV viral load (log_10_)	Undetectable HIV viral load	Good ART adherence
Model of effect of intervention			
**Joint significance tests**	**F(df); p**	**F(df); p**	**F(df); p**
Time	7.8 (3, 791); <0.0001	6.6 (3, 791); 0.0002	3.7 (3, 946); 0.012
Time X intervention	1.3 (4, 791); 0.28	0.95 (4, 791); 0.44	4.4 (4, 946); 0.002
**Interaction effects**	**Beta (SE); p**	**OR (95% CI)**	**OR (95% CI)**
Intervention X Month 2	-0.12 (0.11); 0.29	1.15 (0.72; 1.86)	2.67 (1.64, 4.34)
Intervention X Month 6	-0.15 (0.12); 0.20	1.13 (0.66; 1.92)	1.54 (0.96, 2.46)
Intervention X Month 12	-0.23 (0.12); 0.046	1.58 (0.90; 2.78)	1.19 (0.72, 1.98)
Intervention X Month 18	-0.21 (0.14); 0.15	1.71 (0.77; 3.78)	1.68 (0.97, 2.88)
Model of effect of change in depression status			
**Joint significance tests**	**F(df); p**	**F(df); p**	**F(df); p**
Change in depression status	1.3 (2,456); 0.29	1.5 (2,456); 0.21	1.5 (2,612); 0.31
**Main effects**	**Beta (SE); p**	**OR (95% CI)**	**OR (95% CI)**
Depressed to not depressed	-0.004 (0.08); 0.97	1.33 (0.84, 2.10)	0.78 (0.54, 1.13)
Not depressed to depressed	-0.15 (0.16); 0.35	1.76 (0.70, 4.43)	0.73 (0.36, 1.50)
Repeated not depressed	0.09 (0.10); 0.39	1.01 (0.59, 1.72)	0.95 (0.60, 1.49)
Time (linear)	-0.11 (0.03); <0.0001	1.34 (1.17, 1.52)	1.26 (1.12, 1.41)
Model of effect of change in depressive symptoms (PHQ-9 score)			
**Main effects**	**Beta (SE); p**	**OR (95% CI)**	**OR (95% CI)**
Time (linear)	-0.11 (0.03); <0.0001	1.32 (1.15, 1.51)	1.25 (1.11, 1.40)
Change in PHQ-9 score	-0.002 (0.01); 0.73	0.99 (0.95, 1.02)	0.99 (0.95, 1.02)
Effect size	− 0.012	-0.003	-0.002

ART = antiretroviral therapy; SE = standard error; OR = odds ratio; CI = confidence interval; df = degrees of freedom; PHQ-9 = Patient Health Questionnaire (9-item version)

All models included these baseline covariates: age, relationship status (in a committed relationship; yes/no), any secondary schooling (yes/no), newly diagnosed with HIV (yes/no), PHQ-9 score at eligibility screening, undetectable HIV viral load (yes/no)

The model specification included time as a categorical variable with indicators for each follow-up assessment (month 2, month 6, month 12, month 18). The joint tests of overall effect of time and intervention are Type 3 tests of fixed effects. The coefficients of the main effects of time are not shown here. The key coefficients of interest are the interaction terms between Intervention and time, which are the (covariate-adjusted) difference between the Intervention (intervention = 1) group and the control group (Intervention = 0); a *p*-value < 0.05 indicates a statistically significant difference between the means of the two study arms. Further, note that to estimate the mean in the intervention group, one must add the main effect of time to the interaction between intervention and time. The effect size is a standardized regression coefficient to show magnitude that’s unitless and comparable across models [41].

### Effects on maternal functioning

The intervention had a significant overall effect across the 18-month post-partum follow-up with regards to infant care [F(df) = 14.6 (4,991); *p* <.0001], self-care [F(df) = 3.5 (4,994); *p* =.0075], household functioning [F(df) = 4.8 (4,994); *p* =.001], and post-partum adjustment [F(df) = 5.6 (4,993); *p* =.0002]. Each of these showed a significant intervention effect at one or both of the early post-partum assessments (month 2 and month 6), and only self-care (with a significant intervention effect at each follow-up assessment) and post-partum adjustment showed a significant effect at month 12 or month 18 (see Table 4).

**Table 4:** Linear mixed effects models to evaluate the effects of the M-DEPTH intervention, and change in depression, on measures of maternal functioning over 18 months of post-partum follow-up

	Infant care	Self-care	Household functioning	Work functioning	Overall functioning	Post-partum adjustment
Effect of intervention						
**Joint significance tests**	**F(df); p**	**F(df); p**	**F(df); p**	**F(df); p**	**F(df); p**	**F(df); p**
Time	101.7 (3,991); < 0.0001	78.3 (3,994); <0.0001	273.4 (3,994); <0.0001	6.7 (3,994); 0.0002	350.0 (3,994); <0.0001	8.0 (3,993); <0.0001
Time X intervention	14.6 (4,991); <0.0001	3.5 (4,994); 0.0075	4.8 (4,994); 0.0008	2.1 (4,994); 0.08	1.7 (4,994); 0.16	5.6 (4,993); 0.0002
**Interaction effects**	**Beta (SE); p**	**Beta (SE); p**	**Beta (SE); p**	**Beta (SE); p**	**Beta (SE); p**	**Beta (SE); p**
Intervention X Month 2	0.22 (0.04); <0.0001	0.09 (0.04); 0.03	-0.22 (0.05); <0.0001	-0.01 (0.04); 0.88	-0.02 (0.03); 0.53	-0.03 (0.07); 0.64
Intervention X Month 6	0.17 (0.04); <0.0001	0.11 (0.04); 0.01	-0.05 (0.05); 0.33	0.03 (0.04); 0.45	0.05 (0.03); 0.06	-0.14 (0.07); 0.03
Intervention X Month 12	0.04 (0.04); 0.27	0.10 (0.04); 0.01	-0.03 (0.05); 0.51	0.02 (0.04); 0.69	0.03 (0.03); 0.30	-0.09 (0.07); 0.16
Intervention X Month 18	-0.02 (0.03); 0.53	0.11 (0.03); 0.001	-0.04 (0.04); 0.32	0.08 (0.03); 0.009	0.03 (0.02); 0.18	-0.23 (0.06); <0.0001
Effect of change in depression status						
**Joint significance test**	**F(df); p**	**F(df); p**	**F(df); p**	**F(df); p**	**F(df); p**	**F(df); p**
Change in depression status	84.4 (2,665); <0.0001	63.9 (2,658); <0.0001	0.8 (2,658); 0.44	0.8 (2,658); 0.45	28.2 (2,658); <0.0001	6.9 (2,657); 0.001
**Main effects**	**Beta (SE); p**	**Beta (SE); p**	**Beta (SE); p**	**Beta (SE); p**	**Beta (SE); p**	**Beta (SE); p**
Not depressed to depressed	0.01 (0.05); 0.81	0.04 (0.05); 0.48	-0.06 (0.07); 0.40	0.01 (0.05); 0.86	-0.003 (0.03); 0.93	-0.06 (0.07); 0.40
Depressed to not depressed	0.29 (0.03); <0.0001	0.28 (0.03); <0.0001	-0.04 (0.04); 0.24	0.04 (0.03); 0.21	0.12 (0.02); <0.0001	-0.04 (0.04); 0.24
Repeated not depressed	0.38 (0.03); <0.0001	0.26 (0.03); <0.0001	-0.10 (0.04); 0.03	-0.00 (0.03); 0.99	0.10 (0.02); <0.0001	-0.10 (0.04); 0.03
Time (linear)	0.09 (0.01); <0.0001	0.08 (0.01); <0.0001	0.23 (0.01); <0.0001	-0.02 (0.01); 0.008	0.12 (0.01); <0.0001	0.23 (0.01); <0.0001
Effect of change in depressive symptoms (PHQ-9 score)						
**Main effects**	**Beta (SE); p**	**Beta (SE); p**	**Beta (SE); p**	**Beta (SE); p**	**Beta (SE); p**	**Beta (SE); p**
Time (linear)	0.08 (0.01); <0.0001	0.07 (0.01); <0.0001	0.24 (0.01); <0.0001	-0.03 (0.01); 0.003	0.12 (0.01); <0.0001	0.24 (0.01); <0.0001
Effect of change in PHQ-9 score	-0.03 (0.002); <0.0001	-0.03 (0.002); <0.0001	0.01 (0.003); 0.003	-0.01 (0.002); 0.02	-0.01 (0.001); <0.0001	0.01 (0.003); 0.003
Effect size	- 0.44	− 0.46	− 0.09	− 0.09	− 0.23	− 0.23

SE = standard error; df = degrees of freedom; PHQ-9 = Patient Health Questionnaire (9-item version)

All models included these baseline covariates: age, relationship status (in a committed relationship; yes/no), any secondary schooling (yes/no), newly diagnosed with HIV (yes/no), PHQ-9 score at eligibility screening, undetectable HIV viral load (yes/no)

The model specification included time as a categorical variable with indicators for each follow-up assessment (month 2, month 6, month 12, month 18). The joint tests of overall effect of time and intervention are Type 3 tests of fixed effects. The coefficients of the main effects of time are not shown here. The key coefficients of interest are the interaction terms between Intervention and time, which are the (covariate-adjusted) difference between the Intervention (intervention = 1) group and the control group (Intervention = 0); a *p*-value < 0.05 indicates a statistically significant difference between the means of the two study arms. Further, note that to estimate the mean in the intervention group, one must add the main effect of time to the interaction between intervention and time. The effect size is a standardized regression coefficient to show magnitude that’s unitless and comparable across models [41].

Change in depression status had a significant overall effect across the 18-month post-partum follow-up for infant care [F(df) = 84.4 (2,665); *p* <.0001], self-care [F(df) = 63.9 (2,658); *p* <.0001], overall functioning [F(df) = 28.2 (2,658), *p* <.0001] and post-partum adjustment [F(df) = 6.9 (2,657); *p* =.001]. When looking at levels of the change variable, the effect was significant for each measure among those who went from depressed to not depressed, except for the post-partum measure; all four measures showed a significant effect among those who remained not depressed (see Table 4). Reduction in depressive symptoms over the 18-month post-partum follow-up was significantly associated with each maternal functioning measure, except work functioning (see Table 4); also, effect sizes were medium for infant and self-care.

### Effects on early child development

The intervention had no overall effect across the 18-month post-partum follow-up on any child development measure (see Table 5). Change in depression status had a significant overall beneficial effect on child communication [F(df) = 5.1 (2,660); *p* =.006], and this effect was significant among those whose status went from depressed to not depressed, and those who remained not depressed (see Table 5). The effect of reduced depressive symptoms was significant for better child communication, fine motor functioning, and personal social functioning; although statistically significant, the effect sizes were too small to be practically meaningful (see Table 5).

**Table 5:** Linear mixed effects models to evaluate the effects of the M-DEPTH intervention, and change in depression, on measures of child development over 18 months of post-partum follow-up

	Communication	Gross motor functioning	Fine motor functioning	Problem solving	Personal social functioning	Total functioning
Effect of intervention						
**Joint significance tests**	**F(df); p**	**F(df); p**	**F(df); p**	**F(df); p**	**F(df); p**	**F(df); p**
Time	166.3 (3,998); < 0.0001	39.3 (3,998); <0.0001	55.7 (3,998); <0.0001	200.8 (3,998); <0.0001	99.7 (3,998); <0.0001	109.8 (3,998); <0.0001
Time X intervention	0.24 (4,998); 0.91	3.3 (4,998); 0.01	2.2 (4,998); 0.07	2.9 (4,998); 0.02	0.3 (4,998); 0.89	0.7 (4,998); 0.60
**Interaction effects**	**Beta (SE); p**	**Beta (SE); p**	**Beta (SE); p**	**Beta (SE); p**	**Beta (SE); p**	**Beta (SE); p**
Intervention X Month 2	0.54 (1.40); 0.70	2.64 (1.42); 0.06	1.74 (1.29); 0.18	-2.56 (1.59); 0.11	0.89 (1.38); 0.52	0.89 (5.60); 0.87
Intervention X Month 6	0.75 (1.40); 0.59	-2.66 (1.42); 0.06	-2.63 (1.29); 0.04	-0.36 (1.61); 0.82	-0.67 (1.38); 0.63	-7.74 (5.62); 0.17
Intervention X Month 12	-0.68 (1.42); 0.63	-3.04 (1.44); 0.03	-1.87 (1.31); 0.15	-1.50 (1.60); 0.35	0.88 (1.40); 0.53	-4.94 (5.68); 0.38
Intervention X Month 18	-0.58 (1.41); 0.68	-1.41 (1.43); 0.32	-0.35 (1.29); 0.78	-4.91 (1.59); 0.002	-0.18 (1.39); 0.90	-3.95 (5.63); 0.48
Effect of change in depression status						
**Joint significance test**	**F(df); p**	**F(df); p**	**F(df); p**	**F(df); p**	**F(df); p**	**F(df); p**
Change in depression status	5.1 (2,660); 0.006	0.1 (2,660); 0.89	2.0 (2,660); 0.14	1.8 (2,660); 0.17	3.6 (2,660); 0.03	2.6 (2,660); 0.08
**Main effects**	**Beta (SE); p**	**Beta (SE); p**	**Beta (SE); p**	**Beta (SE); p**	**Beta (SE); p**	**Beta (SE); p**
Not depressed to depressed	2.26 (2.02); 0.26	1.47 (1.82); 0.42	3.17 (1.74); 0.07	5.82 (2.09); 0.006	1.56 (1.74); 0.37	13.69 (7.49); 0.07
Depressed to not depressed	3.41 (1.09); 0.002	0.45 (0.98); 0.65	1.71 (0.94); 0.07	0.94 (1.13); 0.40	2.47 (0.93); 0.008	8.99 (4.02); 0.03
Repeated not depressed	3.57 (1.22); 0.004	1.19 (1.11); 0.28	1.92 (1.08); 0.08	2.34 (1.21); 0.053	2.30 (1.04); 0.03	11.47 (4.52); 0.01
Time (linear)	1.40 (0.36); 0.0001	3.11 (0.32); <0.0001	0.71 (0.31); 0.03	4.76 (0.41); <0.0001	4.87 (0.31); <0.0001	14.84 (1.31); <0.0001
Model of effect of change in depressive symptoms (PHQ-9 score)						
**Main effects**	**Beta (SE); p**	**Beta (SE); p**	**Beta (SE); p**	**Beta (SE); p**	**Beta (SE); p**	**Beta (SE); p**
Time (linear)	1.30 (0.36); 0.0003	3.05 (0.33); <0.0001	0.59 (0.32); 0.06	4.78 (0.41); <0.0001	4.76 (0.31); <0.0001	14.48 (1.32); <0.0001
Effect of change in PHQ-9	-0.28 (0.08); 0.001	-0.09 (0.07); 0.23	-0.20 (0.07); 0.007	0.04 (0.08); 0.60	-0.25 (0.07); 0.0004	-0.78 (0.30); 0.01
Effect size	− 0.10	− 0.07	− 0.09	0.01	− 0.10	− 0.07

SE = standard error; df = degrees of freedom; PHQ-9 = Patient Health Questionnaire (9-item version)

All models included these baseline covariates: age, relationship status (in a committed relationship; yes/no), any secondary schooling (yes/no), newly diagnosed with HIV (yes/no), PHQ-9 score at eligibility screening, undetectable HIV viral load (yes/no)

The model specification included time as a categorical variable with indicators for each follow-up assessment (month 2, month 6, month 12, month 18). The joint tests of overall effect of time and intervention are Type 3 tests of fixed effects. The coefficients of the main effects of time are not shown here. The key coefficients of interest are the interaction terms between Intervention and time, which are the (covariate-adjusted) difference between the Intervention (intervention = 1) group and the control group (Intervention = 0); a *p*-value < 0.05 indicates a statistically significant difference between the means of the two study arms. Further, note that to estimate the mean in the intervention group, one must add the main effect of time to the interaction between intervention and time. The effect size is a standardized regression coefficient to show magnitude that’s unitless and comparable across models [41].

## Discussion

In one of the few controlled trials examining the effects of depression care on maternal and child post-partum health outcomes among WLH in sub-Saharan Africa, the M-DEPTH depression care model showed strong effects on alleviating maternal depression, as well as benefits for maternal functioning, but not early child development outcomes or HIV viral suppression. Similarly, reduced maternal depression was shown to have effects on multiple measures of maternal functioning over the 18-month post-partum follow-up, but not child development, HIV viral suppression or ART adherence.

Our conceptual framework for how treatment for perinatal depression impacts various aspects of the mother’s health and functioning, and the early development of the infant, begins with its effect on perinatal depression (see Fig. 1). Our findings showed that the M-DEPTH depression care model had robust effects on alleviation of depression, both in terms of clinical depression status and reduced symptoms at each post-partum follow-up assessment over 18 months. Nearly all women in the intervention arm who were diagnosed with clinical depression received treatment, and women in the intervention arm were more than 80% less likely to be depressed over the course of the study compared to women in the control group. Evidence-based PST and ADT were implemented by trained and supervised peer mothers and midwife nurses, respectively, highlighting the feasibility and efficacy of using non-specialists to provide care in low resource settings. Other studies have shown the benefits of collaborative care models for perinatal depression [13, 14], but this is among the first conducted in sub-Saharan Africa and among WLH. This is also one of the few studies to establish a durable effect of treatment over 18-months post-partum, as few studies have extended beyond 12 months [43]. 

The next step in the causal pathway of our conceptual model is the impact of alleviated depression on maternal functioning. Consistent with other research [21–23], our data provide support for the benefits of depression treatment on maternal functioning. More specifically, women in the intervention arm reported taking better care of themselves and their infants, and perception of better adjustment to motherhood, compared to women in the control arm. These effects were evident throughout the 18-month post-partum follow-up. Similarly, reduced depression was shown to have effects on these specific aspects of maternal functioning, as well as overall functioning. Together, these findings provide empirical evidence for the benefits of treatment for perinatal depression extending beyond maternal mental health and to impact maternal functioning related to care for self and parenting. Improved mental health may enable a mother to feel more motivated and confident to take care of herself, and to engage with and take care of the needs of her newborn. Problem solving therapy may have been particularly useful in this regard, as it builds skills and confidence to manage stressors and challenges that arise, including those related to parenting.

The benefits of depression care did not extend to early child development outcomes. The intervention had no effect on these outcomes. There was some evidence of reduced depression having benefits for child communication, but effect sizes were low, suggesting that such effects were not likely meaningful. This is consistent with other research that suggests treatment for perinatal depression alone may be insufficient to impact early child development [25], and that supplementary interventions related to parent-child interactions are needed [44]. Furthermore, child health and development are not only influenced by environmental events and interactions with the mother, but also genetic factors that cannot be overcome by improvement in maternal well-being and parenting [45]. 

The intervention and reduced depression did not have any effects on maternal HIV viral suppression, and only a small effect on ART adherence early in the post-partum period, despite depression being a consistent correlate of these aspects of HIV disease management [18, 19]. Prior research in Uganda has suggested that disengagement from HIV and ANC care increases after giving birth [46], but our sample displayed high and consistent levels of ART use and adherence; over half were already fully virally suppressed at baseline, regardless of depression status, and none of the infants newly born to the women in this study tested HIV-positive.

Several study limitations are worth noting. With all participants being stable on ART and exhibiting a consistent HIV/ANC care retention throughout the study, this may have contributed to a ceiling effect and limited the generalizability of our findings. The benefits of depression care may be more evident among women with a greater range of engagement in care, which in turn may be related to increased vulnerability to post-partum depression. Also, the measure of infant development, which relies solely on the ability of the mother to accurately report on infant developmental milestones and observe subtle infant behaviors, may lack the necessary precision to detect intervention effects. Clinician assessment of developmental milestones may have been able to better detect group differences. Lastly, participants were not blinded to treatment condition, so participant responses may have been influenced by social desirability.

## Conclusions

In conclusion, our findings revealed strong durable effects of the M-DEPTH depression care model on alleviating perinatal depression, as well as beneficial effects of the intervention (as well as reduced depression) for maternal functioning—specifically self- and infant care, and post-partum adjustment to motherhood. These benefits of depression care were largely sustained over the 18-month post-partum follow-up; however, these effects did not translate into intervention effects on infant developmental or maternal HIV care adherence and viral suppress. There were some modest effects of reduced depression on better child outcomes, but our findings generally indicate that treatment for maternal depression may not be sufficient to impact early child development. Efforts are needed to increase the availability of depression care for pregnant WLH, but further research is needed to better understand how to augment treatment for perinatal depression with interventions that impact the health and development of the newborn.

## Electronic supplementary material

Below is the link to the electronic supplementary material.

Supplementary Material 1

## Data Availability

De-identified dataset and statistical code are available to researchers upon submission of proposal to G Wagner (gwagner@rand.org) and review by the study team.

## Abbreviations

HIV: Human immunodeficiency virus
WLH: Women living with HIV
M-DEPTH: Maternal Depression Treatment in HIV
ART: Antiretroviral therapy
NIH: National Institutes of Health
PST: Problem solving therapy
ADT: Antidepressant therapy
PMTCT: Prevention of mother-to-child transmission
ANC: Antenatal care
Ush: Uganda shillings
USD: United States dollars
PHQ: Patient Health Questionnaire
FSG: Family Support Groups
SAS: Statistical Analysis Software
Df: Degrees of freedom
SD: Standard deviation
SE: Standard error
OR: Odds ratio
CI: Confidence interval
